# Case Report: CAR-T cell therapy bridging to allogeneic hematopoietic stem cell transplantation triggers Purtscher-like retinopathy: clinical features and complement-mediated microvascular injury mechanisms

**DOI:** 10.3389/fimmu.2026.1670399

**Published:** 2026-01-29

**Authors:** Zhihui Li, Tao Zhang, Qian Fei, Xianxuan Wang, Jing Li, Yong Tao, Tong Wu

**Affiliations:** 1 Department of Bone Marrow Transplantation, Beijing Boren Hospital, Beijing, China; 2 Department of Ophthalmology, Beijing Chaoyang Hospital, Capital Medical University, Beijing, China

**Keywords:** acute leukemia, allogeneic, CAR-T cell therapy bridging, Purtscher-like retinopathy, stem cell transplantation

## Abstract

Purtscher-like retinopathy (PLR) is a secondary, non-traumatic occlusive microvascular retinal disease characterized by retinal leukoderma, hemorrhage, and cotton wool spots. It is commonly associated with conditions such as pancreatitis, renal disease, and infections including COVID-19 but is rarely reported in patients with hematologic malignancies, particularly following hematopoietic stem cell transplantation (HSCT). This article reports a case of relapsed B-cell acute lymphoblastic leukemia (B-ALL) in a patient who underwent multiple lines of immunotherapy, including CD19- and CD22-targeted CAR-T cells, inotuzumab ozogamicin (an anti-CD22 antibody–drug conjugate), and belimumab (a CD19/CD3 bispecific T-cell engager), followed by allogeneic HSCT from an unrelated donor. Early post-transplantation, an influenza A (H1N1) infection likely triggered the onset of PLR. On post-transplant day 160, the patient presented with sudden, painless vision loss in the left eye. Fundoscopic examination revealed retinal hemorrhages, Purtscher flecken, and macular edema, confirming the diagnosis of PLR. By day 194, new-onset thrombocytopenia, proteinuria, and progressively elevated serum creatinine levels suggested an association between PLR and transplant-associated thrombotic microangiopathy (TA-TMA). This case illustrates that multi-agent immunotherapy prior to HSCT for acute leukemia may cause cumulative endothelial injury and that influenza A infection can act as a trigger for PLR in the post-HSCT setting. Early recognition and management of PLR and TA-TMA could improve clinical outcomes. Consequently, close monitoring for these complications is essential in post-transplant patients, particularly those with a history of intensive immunotherapy or subsequent viral infection. Implementing a “systemic-local” endothelial monitoring framework may facilitate timely intervention and enhance patient prognosis.

## 1 Introduction

Purtscher-like retinopathy (PLR) is an ocular condition characterized by sudden vision loss accompanied by retinal ischemia and cotton-wool spots in the absence of trauma. It has been documented in association with acute pancreatitis (1), systemic lupus erythematosus (2), atypical hemolytic uremic syndrome (3), COVID-19 infection (4), and influenza A virus infection (5). In the context of hematologic diseases, TA-TMA remains the predominant cause of retinal complications following hematopoietic stem cell transplantation. However, novel immunotherapies such as CAR-T cell therapy and bispecific antibodies have introduced new pathways of endothelial injury, challenging traditional diagnostic paradigms. Nevertheless, the cumulative endothelial damage resulting from sequential immunotherapies has not been thoroughly investigated, and cases of PLR triggered by post-transplant influenza virus infection are rarely reported.

## 2 Case report

An 11-year-old boy presented to the hematology department in 2017 with pallor. Laboratory tests revealed a white blood cell count of 33.08 × 10^9^/L, hemoglobin of 90 g/L, and a platelet count of 87 × 10^9^/L. Bone marrow morphology demonstrated 78% lymphoblasts. Immunophenotyping identified 92.88% of cells with an abnormal lymphoid phenotype, expressing CD34, CD19, CD10, TdT, cCD79a, HLA-DR, CXCR4, CD58, CD22, CD123, and CD13, with a subset dimly positive for CD38 and negative for CD7, CD117, CD33, CD20, cIgM, and CD15, consistent with a B-cell progenitor phenotype. The *PRAME/ABL1* fusion transcript level was 1.6%, and karyotyping showed normal results. A diagnosis of B-cell acute lymphoblastic leukemia (B-ALL) with a *PRAME* fusion was made. The patient underwent induction chemotherapy with the CODPL regimen. On day 37, a repeat bone marrow assessment showed a *PRAME/ABL1* ratio of 0.28%, with morphologic remission. Flow cytometry detected a minimal residual disease level of 0.003% abnormal immature B cells.

To prevent central nervous system involvement, four prophylactic intrathecal administrations were performed during chemotherapy. All concurrent cerebrospinal fluid analyses, namely, routine, biochemical, and immunophenotypic studies, were normal. The patient subsequently received consolidation therapy with high-dose methotrexate, vindesine, and idarubicin (HD-MTX + VDS + IDR). Reevaluation after this phase confirmed a *PRAME/ABL1* level of 0%, indicating a complete response of the primary disease.

After completing 22 cycles of consolidation therapy, treatment was discontinued. Approximately 2.5 years later, the patient experienced a hematologic relapse. Bone marrow morphology at that time showed 34.5% blasts, and flow cytometry detected 12.62% abnormal immature B cells, with a *PRAME/ABL1* fusion transcript level of 0.19%. Following treatment with murine-derived CD19-targeted CAR-T cell therapy (dose: 4.78 × 10^5^ cells/kg), subsequent bone marrow evaluation confirmed a *PRAME/ABL1* level of 0%, indicating complete remission of the primary disease. The patient then received sequential humanized CD22-targeted CAR-T cell therapy (dose: 2.038 × 10^5^ cells/kg). Posttreatment reevaluation showed a *PRAME/ABL1* level of 0.086%, although bone marrow morphology and flow cytometry results remained normal. This suggested the presence of molecular-level residual disease. However, the patient’s family declined to proceed with hematopoietic stem cell transplantation and elected to be discharged from the hospital.

**Figure 1:**
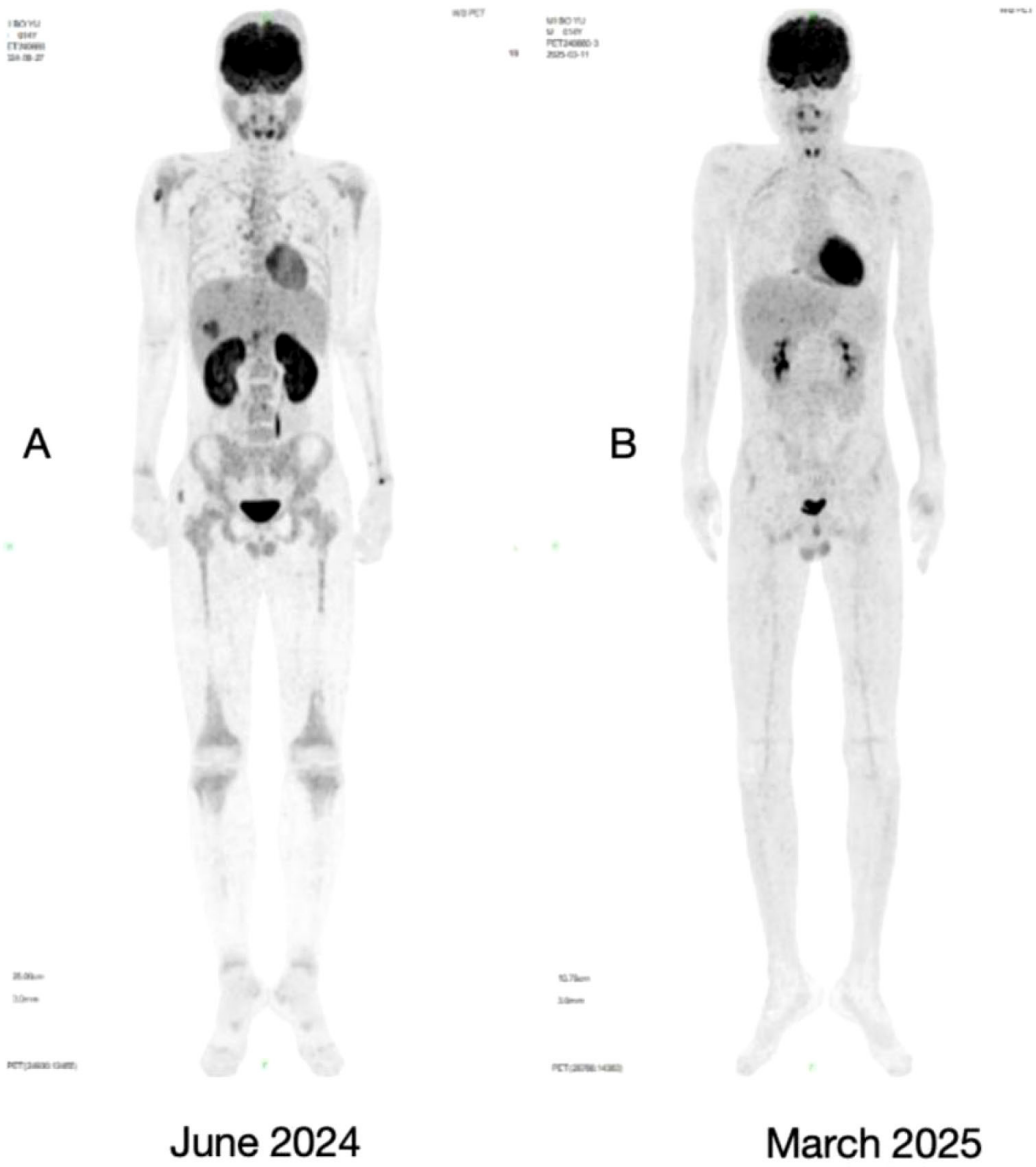
PET-CT examination: Compared to June 2024 **(A)**, the PET-CT in March 2025 **(B)** indicates disease remission status, with extramedullary lesions largely resolved.

In June 2024, a progressively enlarging mass (approximately 6×7 cm) was identified on the left frontal–parietal scalp. A whole-body PET-CT scan demonstrated diffusely increased metabolic activity throughout the skeletal system and bone marrow, consistent with marrow infiltration, along with multiple extramedullary sites of involvement, including the following. (1) Multiple infiltrative foci in the right shoulder, right gluteal musculature, and peritoneal fat. A left frontal–parietal subcutaneous mass exhibited slightly increased density and relatively lower metabolic activity compared to surrounding tissue, suggestive of hemorrhagic soft-tissue infiltration. (2) Widespread lymph node infiltration both above and below the diaphragm, involving bilateral cervical, supraclavicular, mediastinal, hilar, axillary, and multiple retroperitoneal regions. (3) Multiple hepatic infiltrative foci (**Figure 1A**). Bone marrow morphology revealed 94.5% blasts, and flow cytometry detected 97.23% abnormal immature B lymphocytes. Tumor transcriptome sequencing was positive for a *RUNX1-NEK4P1* fusion. Peripheral blood screening for tumor-associated gene mutations identified *PTPN11* p.T73I, *ZBTB7A* p.R377X, and *FOXO1* p.R21C variants. Cytogenetic analysis and cerebrospinal fluid studies were normal. A diagnosis of hematologic relapse with extramedullary disease was confirmed. Initial treatment with inotuzumab ozogamicin resulted in morphologic remission on follow-up bone marrow examination, with flow cytometry detecting 0.06% abnormal immature B lymphocytes. Subsequent therapy with belimumab was followed by repeat molecular testing, which showed that the *PTPN11* p.T73I, *ZBTB7A* p.R377X, and *FOXO1* p.R21C mutations were all below the limit of detection, indicating complete molecular remission of the underlying disease.

The patient subsequently underwent a 10/10 HLA-matched, ABO-identical (donor O+, recipient O+) peripheral blood stem cell transplantation from an unrelated donor on September 11, 2024. The conditioning regimen consisted of total body irradiation (TBI; total dose 10 Gy) combined with etoposide (5 mg/kg/day for 2 days) and the CLAG regimen (cladribine 5 mg/m² daily for 5 days, cytarabine 1 g/m² every 12 h for 5 days, and G-CSF 7.5 μg/kg/day for 5 days). Anti-thymocyte globulin (ATG; thymoglobulin 7.5 mg/kg/day for 4 days) was administered as part of the conditioning. Graft-versus-host disease (GVHD) prophylaxis included cyclosporine, mycophenolate mofetil (MMF), and short-course methotrexate (MTX). Intravenous cyclosporine (1.25 mg/kg every 12 h) was initiated on day −9 and switched to oral formulation 1 month later in the absence of diarrhea. MTX was administered intravenously at 15 mg/m² on day +1, followed by 10 mg/m² on days +3, +6, and +11. MMF (7.5 mg/kg twice daily) was started on day −2, tapered following neutrophil engraftment, and discontinued by day +30.

On day +26, plasma HHV-6 DNA was detected at 1.3 × 10³ copies/mL. Antiviral therapy with foscarnet was added to the immunosuppressive regimen for 3 days, after which the virus became undetectable. On day +29, plasma adenovirus DNA reached 4.3 × 10^5^ copies/mL, and stool adenovirus was 3.6 × 10^8^ copies/mL. The patient received cidofovir and intravenous immunoglobulin for 19 days, after which adenovirus was cleared. Lymphocyte subset analysis on day +30 revealed a CD4/CD8 ratio of 0.22, B cell at 2.26%, and NK cells at 8. 13%. By day +60, the CD4/CD8 ratio was 0.13, with B cell at 3.94% and NK cells at 30.34%.

On day +116, influenza A was detected by RT-PCR from a throat swab and resolved after 1 week of oseltamivir therapy. On day +160, the patient presented to ophthalmology with painless vision loss and metamorphopsia in the left eye. Corrected visual acuity was 0.02 in the left eye and 1.0 in the right eye. Ocular motility, intraocular pressure, and anterior segment examination were unremarkable in both eyes.

**Figure 2:**
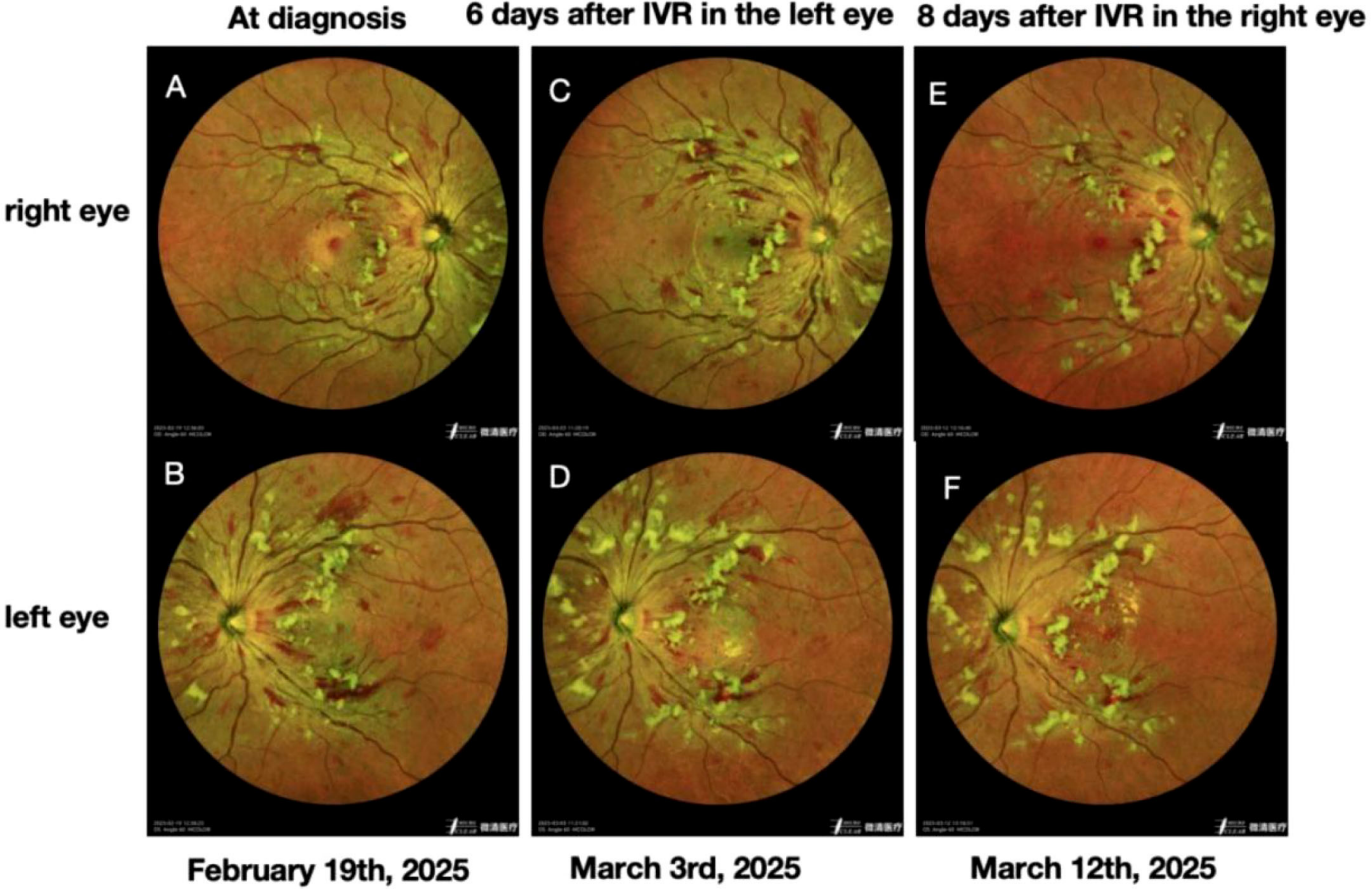
Fundus images of both eyes. **(A, B)** Multiple retinal hemorrhages, cotton-wool spots, and yellowish-white Purtscher flecken are visible in the posterior pole of both eyes, with more extensive involvement in the left eye. **(C)** The right eye shows a marked increase in retinal hemorrhages, cotton-wool spots, and Purtscher flecken compared to earlier observations. **(D)** Six days after intravitreal ranibizumab injection in the left eye, retinal hemorrhages have partially absorbed; cotton-wool spots and Purtscher flecken remain largely unchanged. **(E)** Eight days after intravitreal ranibizumab injection in the right eye, retinal hemorrhages, cotton-wool spots, and Purtscher flecken show noticeable regression. **(F)** 15 days after intravitreal ranibizumab injection in the left eye, further resolution of retinal hemorrhages is evident, along with reduction in cotton-wool spots and Purtscher flecken.

**Figure 3:**
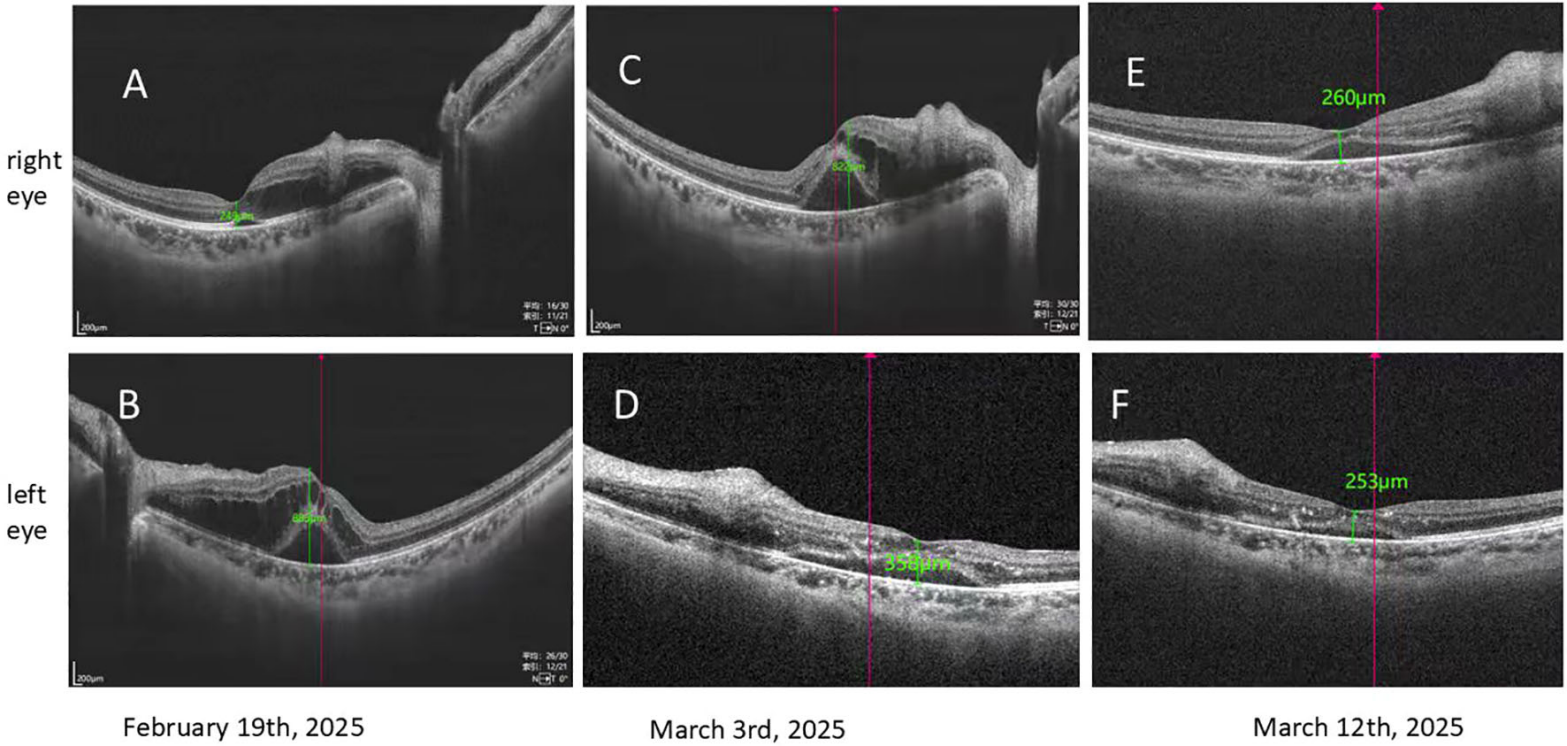
Optical coherence tomography images of both eyes. **(A)** Right eye (February 19): Edema and thickening of the retinal inner layers, with cystoid macular edema measuring approximately 249 μm in thickness. **(B)** Left eye (February 19): Edema and thickening of the retinal inner layers, with cystoid macular edema measuring approximately 885 μm. Multiple focal hyperreflective foci are present. **(C)** Right eye (March 3): Worsening edema and thickening of the retinal inner layers, with cystoid macular edema measuring approximately 822 μm, increased compared to prior imaging. **(D)** Left eye (March 3): Reduced edema and thickening of the retinal inner layers, with cystoid macular edema measuring approximately 358 μm, decreased compared to prior imaging. **(E)** Right eye (March 12): Improvement in edema and thickening of the retinal inner layers, with cystoid macular edema measuring approximately 253 μm, significantly reduced compared to earlier examinations. **(F)** Left eye (March 12): Cystoid macular edema measuring approximately 260 μm, showing minimal change compared to prior imaging.

**Figure 4:**
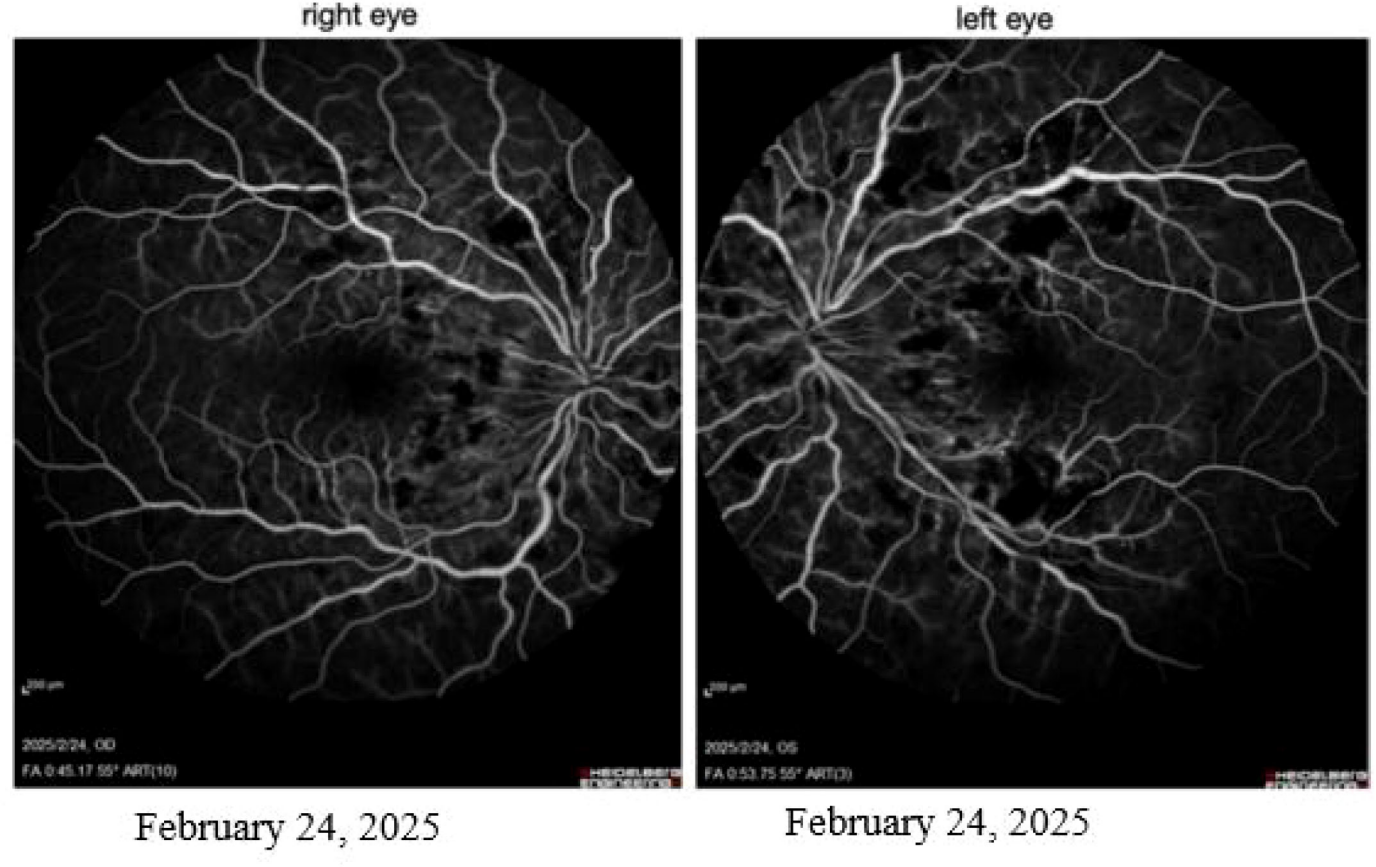
Fluorescein angiography of both eyes demonstrates extensive punctate leakage and irregular patchy non-perfusion areas in the peripapillary and macular regions.

Both eyes exhibited vitreous opacities. Fundus photography revealed flame-shaped hemorrhages in the posterior pole bilaterally, with macular involvement in the left eye (**Figures 2A, B**). Optical coherence tomography demonstrated macular edema, which was more pronounced in the left eye (**Figures 3A, B**). Fluorescein angiography showed extensive non-perfusion areas surrounding the optic disc and within the macular region (**Figure 4**). A systemic PET-CT scan showed no evidence of extramedullary infiltration (**Figure 1B**), ruling out relapse of the underlying malignancy.

In the absence of trauma, a diagnosis of PLR was made. The patient commenced treatment with intravitreal injections of ranibizumab (0.5 mg) in the left eye on a monthly schedule. Six days after the first injection, visual acuity in the left eye showed marked improvement (**Table 1**), with corresponding reductions in optic disc edema and retinal hemorrhage (**Figures 2D**, **3D**).

**Table 1:** Changes in binocular corrected vision.

Date	Treat	Left eye visual acuity (corrected)	Right eye visual acuity (corrected)
2025-2-13		0.12	1.0
2025-2-19		0.02	1.0
2025-2-25	Lezumab 0.5 mg was injected intracapsular in the left eye		
2025-3-3		0.3	0.15
2025-3-4	Ranibizumab 0.5 mg was injected intracapsular in the right eye		
2025-3-12	Lezumab 0.5 mg was injected intracapsular in the left eye	0.6	0.6
2025-3-25	Lezumab 0.5 mg was injected intracapsular in the left eye	0.6	0.6

Analysis of aqueous humor by cytometric bead array (CBA) revealed elevated levels of bFGF (7.8 pg/mL; reference <1.0 pg/mL) and VCAM-1 (2212.9 pg/mL; reference 200–1,000 pg/mL), indicating compromise of the blood–retinal barrier. However, during this period, visual acuity in the right eye deteriorated rapidly (**Table 1**), accompanied by a significant increase in retinal hemorrhage and cotton-wool spots (**Figure 2C**) and worsening optic disc edema (**Figure 3F**). Intravitreal ranibizumab (0.5 mg) was subsequently initiated in the right eye. Eight days after the right eye received its first injection, visual acuity in the left eye continued to improve. Although visual acuity in the right eye did not show further gain (**Table 1**), fundus examination revealed significant improvement in retinal hemorrhages, cotton-wool spots, and papilledema in both eyes (**Figures 2E, F**, **3E, F**).

Day +179: Lymphocyte subset analysis revealed a CD4/CD8 ratio of 0.22, a B-cell proportion of 5.05%, and an NK cell proportion of 21.68%, indicating impaired immune reconstitution.

Day +194: The patient had developed severe thrombocytopenia (platelet nadir 40 × 10^9^/L), nephrotic-range proteinuria (24-h urinary protein 2,400 mg), deterioration of renal function (serum creatinine 133.9 μmol/L), and complement activation (C5b-9–298 ng/mL), with multi-compartment serous effusions evident on imageological examination.

GVHD was excluded based on the absence of rash, diarrhea, or significant transaminitis. Calcineurin inhibitor toxicity was ruled out (cyclosporine concentration <10 ng/mL after day +150; cyclosporine was discontinued on day +180, with steroids initiated for GVHD prophylaxis). Schistocytes were present at 0.8%.

A diagnosis of TA-TMA was made, and treatment with dipyridamole and etanercept was initiated. Supportive measures including diuresis, antihypertensive therapy, and management of capillary leak provided symptomatic relief, and the patient’s condition remained generally stable thereafter.

The timeline of key clinical events in the above medical history is shown in **Tables 2**, **3** below.

**Table 2:** Key clinical events before transplantation.

Date	Pre-transplant events	Treat	Bone marrow morphology	Bone marrow flow	Gen
2017-09-01	Make a definite diagnosis B-ALL		78%	92.88%	PRAME/ABL=1.6%
2017-09-02		Induction chemotherapy with CODPL	No abnormality seen	0.003%	PRAME/ABL=0.28%
2017-10-04		HDMTX+VDS+IDR	No abnormality seen	0%	PRAME/ABL=0%
2017-11-01~2029-05-01		HDMTX+VDS*10 cycles, HDMTX+VDSHIDR*1 cycle, HDAra-C+IDR2 cycles, HDMTX+VDS+Peg “2 cycles, IDR+VDS” 2 cycles, CTX+VDS+Peg 1 cycle, CTX+VDS*1 cycle, HDAra-c*1 cycle, IFO+VDS+VP-16*1 cycle, maintenance 22 chemotherapy	No abnormality seen	0%	PRAME/ABL=0%
2021-12-27	Recurrent disease hematological		34.5%	12.62%	PRAME/ABL=0.19%
2021-12-23		CD19-CAR-T	No abnormality seen	0%	PRAME/ABL=0%
2022-01-25		CD22-CAR-T	No abnormality seen	0%	PRAME/ABL=0.086%
2024-06-06	The disease was fully recurrent with extramedullary infiltration		94.5%	97.23%	RUNX1-NEK4P1 positive; PTPN11 p.T73I, ZBTB7Ap.R377X, FOXO1 p.R21C gene mutation
2024-07-03		Ogai trastuzumab	No abnormality seen	0.06%	
2024-08-13		Belimumab	No abnormality seen	0%	PTPN11 p.T73I, ZBTB7Ap.R377X, FOXO1 p.R21C gene mutations were quantitatively negative

**Table 3:** Key clinical events after transplantation.

Date	Post-transplant events	Treat	Bone marrow morphology	Bone marrow flow	Gen	Inoculants
2024-09-11/2024-9-12	HSCT					
2024-10-08 (+26d)	HHV6 positive	The virus turned negative after immunosuppressive agent treatment				
2024-10-11 (+29d)	ADV positive	Sodifavir and gamma globulin treatment turned negative	No abnormality seen	0%	0%	Full donor type
2024-11-11 (+65d)			No abnormality seen	0%	0%	Full donor type
2024-12-15 (+94d)			No abnormality seen	0%	0%	Full donor type
2025-01-06 (+116d)	Positive for influenza A	Oseltamivir treatment turned negative				
2025-01-13 (+123d)			No abnormality seen	0%	0%	Full donor type
2025-2-10 (+151d)			No abnormality seen	0%	0%	Full donor type
2025-02-19 (+160d)	PLR	The patient improved after injection of lezumab in both eyes				
2025-3-10 (+179d)			No abnormality seen	0%	0%	Full donor type
2025-03-25 (+194d)	TA-TMA	The condition was basically stable after the removal of vincoside and icuzumab				

## 3 Discussion

Purtscher retinopathy (PuR) was first described by Otmar Purtscher in 1910^6^ (6). He observed that patients without direct ocular trauma could develop visual loss after head injury, presenting with tortuous retinal veins, cotton-wool spots near the macula, and occasional flame-shaped or linear hemorrhages. In recent years, similar fundus findings have been identified in the absence of external trauma. This clinical entity is collectively termed Purtscher-like retinopathy (PLR) and has been associated with various systemic conditions such as acute pancreatitis (1) and systemic lupus erythematosus (2), among others.

Reports of PLR in acute leukemia patients following hematopoietic stem cell transplantation (HSCT) are rare. Paul Castillol et al. (7) described a case of relapsed/refractory B-cell acute lymphoblastic leukemia treated with myeloablative conditioning (total body irradiation, cytarabine, cyclophosphamide, and alemtuzumab) followed by allogeneic HSCT. Transplant-associated thrombotic microangiopathy (TA-TMA) was diagnosed on day +164 post-transplant and managed with plasma exchange and etanercept. PLR was confirmed on day +188 due to blurred vision, with significant visual improvement after 5 weeks of intravitreal triamcinolone acetonide injections. In this first report, we describe a case of relapsed B-ALL in which PLR developed after HSCT, preceded by multiple lines of immunotherapy, and was likely triggered by influenza A virus infection. TA-TMA is a severe complication of HSCT, characterized by microangiopathic hemolytic anemia, thrombocytopenia, microvascular thrombosis, and multiorgan dysfunction, and carries a high mortality rate. This case is notable for two reasons. First, the patient received multiple immunotherapies prior to HSCT, which may have caused cumulative endothelial damage and increased susceptibility to post-transplant TA-TMA. Second, TA-TMA in this patient presented initially as PLR following influenza A infection, a pattern differing from previous reports and suggesting distinct clinical forms of “systemic-local” endothelial injury. Immunotherapy is known to induce endothelial dysfunction. CAR-T cell therapy can trigger macrophage activation and cytokine release syndrome (8), leading to a surge in cytokines such as interferon-γ, IL-1, IL-6, TNF, and IL-18. Cytokines including IL-6 and TNF-α directly activate vascular endothelial cells, disrupting barrier function and increasing vascular permeability (9, 10). Belimumab is a bispecific T-cell engager that binds CD3 on T cells and CD19 on B cells, activating T cells to lyse tumor cells. Klinger et al. (11) found that belimumab induces T-cell redistribution by promoting a conformational change in LFA-1 to an intermediate-affinity state, enhancing binding to ICAM-1/Fc chimeric protein and promoting T-cell adhesion to the vascular endothelium, thereby contributing to endothelial dysfunction. Our patient had previously received three distinct immunotherapies, supporting the likelihood of preexisting endothelial injury before transplantation.

It is also notable that the CD22-targeted antibody–drug conjugate inotuzumab ozogamicin induces B-cell depletion. The time to B-cell recovery varies unpredictably among patients, leading to prolonged humoral immunodeficiency and an increased risk of viral infection (12). In this case, serial post-transplant lymphocyte subset monitoring showed that the B-cell proportion never exceeded 6% (by flow cytometry), reflecting absent B-cell immune function and consistent humoral immunodeficiency. This likely contributed to the sequential early post-HSCT infections with adenovirus (ADV), HHV-6, and H1N1, each of which cleared following appropriate antiviral therapy.

Pre-transplant conditioning, which typically involves chemotherapy and radiation, especially total body irradiation (TBI), can disrupt vascular endothelial homeostasis and cause endothelial injury. For example, radiation directly activates and damages the vascular endothelium through mechanisms such as upregulation of adhesion molecules, enhanced leukocyte–endothelial interactions, mitochondrial impairment, increased barrier permeability, and apoptosis (13). Additionally, post-transplant medications used for graft-versus-host disease (GVHD) prophylaxis, including calcineurin inhibitors and mTOR inhibitors, can markedly elevate endothelial release of prostacyclin and thromboxane A_2_, potentially worsening endothelial damage via vasoconstriction and microthrombus formation (14).

Furthermore, although the recipient’s immune system is fully replaced by donor-derived cells after allogeneic hematopoietic stem cell transplantation, the vascular endothelium continues to be of host origin. If the patient has preexisting endothelial abnormalities, the stability of the endothelial compartment may remain compromised after transplant, rendering it more vulnerable to thrombotic microangiopathy in the setting of insults such as infection or rejection (15).

TA-TMA is a rare but serious complication following hematopoietic stem cell transplantation. A retrospective study by Ramgopal et al. (16) identified significant associations between TA-TMA and HSCT, cytomegalovirus (CMV), HHV-6, fungal infections, and graft-versus-host disease (GVHD) (p = 0.01). Influenza-associated TA-TMA is less frequently reported; prior studies indicate that influenza virus can elevate levels of inflammatory mediators such as TNF and IL-6, increase vascular permeability, and thereby mediate endothelial barrier dysfunction, leading to thrombotic events (17). During the H1N1 pandemic, Bunce et al. (18) reported a retrospective series of 119 patients in which four venous thromboembolic and three arterial thromboembolic events occurred. Similarly, Bitzan et al. (19) described 30 cases of TA-TMA, 25 of which were linked to influenza virus infection and the remaining 5 to influenza vaccination. Influenza-related TA-TMA may be triggered by underlying complement gene deficiencies and/or the presence of autoantibodies against factor H (CFH) or ADAMTS13. In the present case, germline predisposition gene screening prior to transplantation revealed that the patient carried a class II variant in *TTC37*, associated with predominantly antibody deficiencies. The patient had received multiple lines of immunotherapy and underwent a myeloablative conditioning regimen based on total body irradiation (TBI), likely resulting in cumulative endothelial injury. Poor early post-transplant humoral immune reconstitution led to successive viral infections, collectively increasing susceptibility to TA-TMA compared to other transplant recipients.

In summary, the patient experienced multiple endothelial insults following a history of sequential treatments, including CAR-T cell therapy, bispecific antibody exposure, myeloablative conditioning, and calcineurin inhibitor use, as well as infections with HHV-6, adenovirus, and influenza virus, which collectively led to the development of PLR. We present this rare case to raise clinical awareness of the diagnosis and management of PLR and TA-TMA in the post−transplant setting, with the goal of facilitating timely intervention and improving patient outcomes.

## Data Availability

The original contributions presented in the study are included in the article/supplementary material. Further inquiries can be directed to the corresponding authors.
